# Perceived benefits and challenges of school feeding program in Addis Ababa, Ethiopia: a qualitative study

**DOI:** 10.1017/jns.2024.42

**Published:** 2024-09-18

**Authors:** Yihalem Tamiru, Afework Mulugeta, Abebe Ayelign, Dube Jara, Elyas Melaku, Samson Gebremedhin

**Affiliations:** 1 Center of Food Science and Nutrition, College of Natural and Computational Sciences, Addis Ababa University, Addis Ababa, Ethiopia; 2 Department of Public Health, College of Health Sciences, Mekelle University, Mekelle, Ethiopia; 3 School of Public Health, College of Health Sciences, Addis Ababa University. Addis Ababa, Ethiopia; 4 Department of Public Health, College of Medicine and Health Science, Debre Markos University, Debre Markos, Ethiopia

**Keywords:** Addis Ababa, Benefits, Challenges, Ethiopia, Home-Grown School Feeding Program

## Abstract

Addis Ababa initiated a universal Home-Grown School Feeding Program (HGSFP) in February 2019 to address hunger and improve the educational outcomes of schoolchildren. This study aimed to document the perceived benefits and challenges of the HGSFP in Addis Ababa, where such information was lacking. In May 2023, a qualitative phenomenological study was conducted to collect data from 20 schools participating in the HGSFP. Data were collected through key informant interviews and focus group discussions (FGDs) involving 98 purposively selected participants. The study encompassed 48 student mothers in 5 FGDs, 20 student interviews, 20 school principals, and 10 experts from the Ministry of Education, Sub-cities, and the School Feeding Agency for in-depth interviews. Data collected in the local language were transcribed, translated into English, and thematically analysed using ATLAS-TI software. The study’s findings unveiled the transformative impact of the HGSFP in Addis Ababa, Ethiopia. It demonstrated remarkable improvements in attendance, concentration, academic performance, reduced dropout rates, financial relief, enhanced behaviour, and a safer learning environment. However, urgent measures are imperative to tackle pressing challenges such as underpaid kitchen workers, operational issues, reduced reading time, rising food costs, limited market access, inadequate infrastructure, and growing dependency. To ensure the enduring sustainability of HGSFP, addressing challenges like workload reduction, kitchen infrastructure enhancement, government guideline implementation, promoting self-reliance, overcoming budget limitations, and addressing school gardening obstacles is vital.

## Introduction

The school feeding program is one of the world’s largest and most widespread social safety net programs, benefiting nearly half a billion school children worldwide.^(1)^ An estimated 66 million primary school children, of which 23 million are in Africa, attend school hungry, struggle to learn, have poor concentration, and have little interest in learning.^(2–6)^ Furthermore, approximately 67 million children do not attend school at all.^(7)^ Attending classes while hungry hurts children’s and adolescents’ ability to learn, thrive, and reach their full potential.^(6,8)^

School feeding programs (SFPs) are widely regarded as a game-changing option for improving food availability and education, as well as a prominent and innovative vehicle for addressing multiple Sustainable Development Goals (SDGs) outcomes.^(1,9)^ SFPs help school-age children and adolescents develop physically, mentally, and emotionally, especially in low- and middle-income nations.^(6)^ The World Bank and the World Food Program (WFP) published a joint review of SFPs in 2009^(10)^ reinforcing the rationale and objectives of SFPs. The three main goals identified were to provide safety nets for families to absorb social and economic shocks, to improve school-aged children’s education and scholastic performance, and to improve children’s nutrition and health status.^(10)^

Malnutrition impairs the academic performance of schoolchildren from low-income families.^(11)^ A 2015 study in Ethiopia found that malnutrition affected 31% of schoolchildren (19.6% stunted, 15.9% underweight, and 14.0% wasted).^(12)^ Furthermore, studies have shown that adolescent girls and boys aged 15–19 years are prone to chronic energy deficiency.^(11,13)^

School feeding in Ethiopia began in 1994 as a pilot project by the WFP in war-affected zones in Tigray Region.^(14–16)^ Later, the aid was expanded to the remaining five food-insecure regions in the Somali Regional States: Afar, Amhara, Oromia, and SNNPR.^(16)^ Currently, in Ethiopia, homegrown SFPs are run by locally produced food products.^(17)^ From the perspective of the WFP, HGSFPs aim to both increase children’s well-being and promote local agricultural production and development by providing an ongoing market for small landholders.^(18)^

The program is implemented through three approaches: government-owned and financed homegrown school feeding programs (HGSFPs), NGO-run SFPs like the WFP, and community-initiated and owned SFPs. It is supported by national school feeding policy, food and nutrition policy, and strategy.^(19,20)^ The program also receives political attention and support from various stakeholders.

In Addis Ababa, the HGSFP was initiated on a small scale by charitable societies, NGOs, and other stakeholders,^(11,21)^ and now the city administration has expanded the program and assumed full ownership. Earlier, economically disadvantaged students used to be targeted by the program; however, since February 2019, the program has covered all public pre-primary and primary schools (grades 1–8) in the city.^(11,21)^ Ethiopia’s government has made every effort to ensure that all of the country’s children have access to education. In order to increase learning achievement, reduce temporary hunger, and enhance the health and nutritional status of schoolchildren, the Addis Ababa City Administration School Feeding Agency is currently implementing HGSFPs.^(11,21)^

Furthermore, little is understood about the benefits and challenges of HGSFPs in Ethiopia. Research conducted in the Sidama Region, Southern Ethiopia, and the Somali Regional State revealed several challenges.^(14,22,23)^ These include the absence of a consistent supply of clean water, the delay in the delivery of rations, poor quality food provision, the insufficient amount of food allotted for the academic year, the lack of program infrastructure, the lack of sanitation and hygiene training for cooks, inadequate funding for schools and independent structures, inconsistent resource mobilisation, ineffective monitoring and evaluation, and inappropriate use of the allotted food.^(14,22,23)^ Another study carried out in the Jigjiga Zone, Somali regional area, Ethiopia, revealed that the main program challenges were water supply, storage facility shortages, kitchen utensil issues, and delays in ration delivery.^(23)^ The benefits include raising students’ academic performance and attendance in class. Additionally, the program helped the parents save money and time.^(23)^

Several donors and national governments from both developed and developing countries have also invested millions of dollars in SFPs.^(24,25)^ Despite the attention and resources devoted to SFPs, little rigorous evidence exists to support these investments,^(25)^ and no adequate research on HGSFPs has been conducted in our country to date,^(24,26)^ and there is a lack of studies that directly address the unknown perceptions of parents, school principals, students, and other stakeholders towards HGSFPs.^(27)^

Our study focuses on the HGSFPs in Addis Ababa, the capital city of Ethiopia. This urban setting presents unique characteristics that contribute to our understanding of HGSFP. By examining the perceived benefits and challenges of urban HGSFP, our research informs policy frameworks and implementation strategies not only in Addis Ababa but also in other urban areas. Through qualitative methods like interviews and focus groups, we gather diverse perspectives on the perceived benefits and challenges of HGSFP in public primary schools. Our findings have broad applicability, filling a crucial literature gap and contributing to the fields of nutrition and education.

## Materials and method

### Study setting and period

The study was conducted in twenty primary schools in five selected sub-cities in Addis Ababa, the capital city of Ethiopia,^(28)^ from 10 April 10 to 26 May 2023. Addis Ababa is the country’s largest city and plays an important political, economic, and symbolic role in the country.^(28)^ By 2036, the population is expected to exceed 5 million, as reported by the Central Statistical Agency.^(28)^ In 2019, the City Administration School Feeding Agency employed 10,000 mothers from various unions to participate in the school feeding program. Each mother was responsible for preparing meals for a group of 30–50 children.^(15)^ The HGSFP has now been implemented in all 264 public primary schools located in all 11 sub-cities, and the city government provides a school feeding program for about 638,857 students.^(29)^

### Study population

- Mothers of beneficiary students from 20 schools participating in the HGSFP.
- Grades 6 and 7 students enrolled in the HGSFP participating schools.
- Public primary school directors directly involved in implementing, coordinating, or managing the HGSFP in the selected schools.
- Experts from the Ministry of Education, Sub-cities, and the School Feeding Agency were responsible for overseeing, coordinating, planning, executing, supervising, and managing various aspects of the HGSFP.

### Inclusion criteria

- Mothers of beneficiary students actively participating in the HGSFP.
- Grades 6 and 7 students in HGSFP-participating schools, who are capable of conducting independent interviews.
- School administrators or directors directly involved in the implementation, coordination, or management of the HGSFP.
- Ministry of Education experts, sub-city experts, and School Feeding Agency experts involved in oversight, coordination, planning, execution, supervision, and operational aspects of the HGSFP.

### Exclusion criteria

- Individuals who are not mothers of beneficiary children in the HGSFP.
- Individuals who are not actively engaged in the HGSFP or do not have direct responsibilities related to its implementation.
- Students who have opted out or declined to participate, and those with significant physical or mental health conditions affecting interview accuracy.

### Study design

This study employed a quantitative phenomenological study with a case study approach to examine the challenges and perceived benefits of SFPs in the public primary schools of Addis Ababa city. A case study is a qualitative research method that enables an in-depth examination of a phenomenon or a program using a variety of data sources in its natural context.^(30,31)^

### Theme selection

The selection of themes for our study on the HGSFP was a meticulous process guided by multiple criteria. We considered our research objectives, feasibility, relevance, significance, stakeholder input, ethical guidelines, and resource availability. These considerations ensured a comprehensive exploration of the program within the limitations of our study. By addressing these criteria and considerations, we conducted a rigorous analysis and generated meaningful insights into the HGSFP.

### Obtaining parental consent

To obtain parental consent, we employed strategies such as parent-teacher meetings, existing platforms, and in-person discussions led by school directors. We explained the study’s purpose, procedures, risks, benefits, and confidentiality measures in detail. Consent forms were carefully explained, and parents were encouraged to ask questions and express concerns. Our procedures followed institutional and ethical guidelines, guaranteeing data security, anonymity, and confidentiality. These strategies successfully obtained parental consent while honoring their autonomy.

### Sampling procedure

A multi-stage sampling procedure was employed to select the participants for the study. Three rounds of sampling were used to choose the final research subjects. In the first step, five of the eleven sub-cities (or 50% of the sub-cities) were selected using a lottery technique. Arada, Bole, Kirkose, Ledeta, and Yeka Sub-city were the five sub-cities that were selected. In the second phase, after identifying each sub-city’s public primary school, four were selected at random from each sub-city. In the study, twenty primary schools with HGSF programs were included.

In consultation with the school principals, all study participants who had major roles in the HG-SFPs (parents, students, SFP coordinators, and school directors) were specifically chosen. Furthermore, the current study purposefully included experts from the sub-cities, the Addis Ababa City Administration School Feeding Agency, and the Ministry of Education.

The study involved five parent focus groups, twenty key informant interviews with SFP coordinators and school directors, and interviews with twenty beneficiary students. Moreover, in-depth interviews were conducted with three experts each from the Ministry of Education and the Ababa City Administration School Feeding Agency, along with four experts from the sub-city.

We decided the number of study participants based on the study’s design, the population’s diversity, and the depth of the data. Many academics advise using a range of research participant sizes. For example, Lincoln and Guba advise 12–20 participants for studies based on interviews.^(32)^ Considering these factors, the key informants in this study were purposefully selected. There were 48 participants in all for the focus group discussions (FGDs); each FGD had an average of ten participants. According to data saturation principles, which defined the point at which additional interviews produced no new data, the study’s final sample size was 98 participants.^(33)^

### Sampling strategy

To comprehensively analyse the HGSFP, we employed a sampling strategy by selecting four schools from each of Addis Ababa’s five sub-cities. This approach captures variations in program implementation, contextual factors, and stakeholder perspectives, enhancing the reliability and generalisability of our findings. We considered practical factors like socioeconomic, demographic, and geographic characteristics to understand the factors influencing the HGSFP’s success or limitations within each sub-city. Our strategy encompasses diverse school characteristics, stakeholder perspectives, and potential variations in challenges and commitment-related issues across sub-cities. It acknowledges the unique challenges and commitment levels faced by schools in different sub-cities due to socioeconomic factors, population density, and infrastructure. Including multiple schools from each sub-city provides a comprehensive understanding of challenges and commitment-related issues from various stakeholders, including students, parents, school directors, program administrators, and experts. This strategy also enables us to explore intra-sub-city variations resulting from differences in school demographics, resources, and community involvement.

### Participant selection

To achieve a comprehensive understanding of our research topic, we selected participants from diverse households, including individuals with varying family backgrounds, socioeconomic statuses, and living conditions. This approach enhances the representativeness of our sample and improves the validity and reliability of our data by incorporating independent perspectives from students and parents. Furthermore, the inclusion of participants from diverse households increases the generalisability of our findings to a broader population. Although establishing a direct link between specific family characteristics and student experiences or outcomes may have limitations, we employ statistical techniques and control for relevant variables to extract valuable insights. This approach aligns with our research objectives, capturing a wide range of perspectives and facilitating a comprehensive understanding of the factors under study.

### Data collection tools and procedure

Semi-structured interviews and FGDs guides were developed to assess the challenges and perceived benefits of the homegrown school feeding program in the study area. Before being used for the actual data collection, these guides were developed by reviewing various literature and evaluated by three experts in the field. The guide was prepared in the English language following the review of literature.^(31,34–36)^

Data for this exploratory qualitative study was collected from twenty primary schools that implemented HGSFPs. The data collection involved key informant interviews, FGDs, beneficiary student interviews, in-depth interviews with school principals, and interviews with experts from the Ministry of Education, Addis Ababa City Administration, School Feeding Agency, and sub-cities. The interviews took place in private settings, and audio recordings were made in Amharic (the national language) with the participants’ consent.

Data were audio-taped and transferred to a personal computer to which only investigators had access. Audio-taped files were transcribed verbatim using the local language and translated into English. Each FGD and KII was transcribed before the next data collection, which enabled the capture of emerging insights into the semi-structured guide to enhance the credibility and comprehensiveness of the conversations. The principal investigator conducted a triangulation of the data generated from FGDs and KIIs. The principal investigator has experience gathering qualitative data and has taken advanced qualitative research methodologies.

Data was collected using pre-tested, semi-structured, in-depth interview guides. These guides included predetermined questions and prompts, allowing flexibility in participants’ responses. The interviews occurred in relevant locations like schools and government offices, chosen for participant convenience and a comfortable environment.

The data collection team comprised five individuals with a master’s degree in public health and nutrition, experienced in qualitative inquiries. After a three-day training session covering study objectives, data collection techniques, and ethical principles, the trained data collectors conducted in-depth interviews. FGDs were moderated by the principal investigator and a note-taker. Interviews with school principals and stakeholders from the Ministry of Education, sub-cities, and the school feeding agency took place in the mornings at their respective offices.

To address the potential influence of work-related exhaustion on data quality, interviews with principals were scheduled in the morning hours, taking into account our natural productivity rhythms. All respondents engaged in face-to-face interviews, while in-depth interviews with principals were conducted in their respective offices, lasting approximately forty to sixty minutes. Furthermore, five FGDs with parents of students were held in a private conference room within the school, with each session spanning between 60 and 90 minutes.

To reduce socially desirable responses to interview questions, all of the student interviews took place in a separate room with just the interviewee present. After reaching a saturation level, interviews with students were stopped. An average of ten parents of students participated in each focus group discussion (n = 48; 10, 10, 9, 9, and 10 participants) and ranging from 6 to 12 participants.^(31)^

### Data quality assurance

Appropriate note-taking and abstraction were carried out to maintain the quality of the data. The data’s credibility and dependability were maintained through continuous follow-up and data triangulation in time, person, and place.

### Reflexivity

Reflexivity was employed to increase the data collection’s rigor. This made it possible to conduct interviews with better probing, fewer assumptions, avoidance of early interpretation, and an amplified feeling of curiosity. To increase engagement and trust, share interview control, and ultimately increase the richness of the interview content, we also used reciprocity between the interviewer and interviewee as a technique.

### Data analysis

Data collection and analysis commenced simultaneously. The verbatim Amharic transcriptions of the audio files were translated into English following their initial translation into Amharic for the analysis. The theme analysis method recommended by Braun and Clark^(37)^ was employed, which involved a thorough examination of the transcripts using the theoretical lens of the resilience framework.^(37,38)^

The transcripts were independently read and reread by two authors to gain a comprehensive understanding of the data and generate initial codes. Thematic analysis was conducted by grouping related codes into categories and categories into themes. The software Atlas IT was utilized to analyse the data and develop a final coding scheme based on emergent themes. As new themes emerged, existing themes were modified and added. Through multiple discussions, the research team refined, mapped, and organized the codes into themes. These themes underwent further refinement until a consensus was reached on their interpretation and meaning. The draft findings were shared with culturally competent academics and stakeholders for validation and deeper contextual insights. The analysis concluded after thorough discussions between the authors.

### Trustworthiness and rigor

Lincoln and Guba developed strict standards for determining trustworthiness in qualitative research, known as credibility, dependability, confirmability, and transferability.^(39–41)^ Different measures were taken to ensure the trustworthiness of these findings, including participant triangulation (data were gathered from students, parents, and teachers), a method of triangulation (in-depth interviews, FGDs, and document reviews), and extended engagement to build rapport and trust among participants. Expert-reviewed interviews and FGD guides were employed.

Credibility was addressed using a variety of approaches, such as prolonged involvement, persistent observation, data collection, and researcher triangulation. Frequent peer debriefing was carried out to assess referential adequacy, check preliminary findings and interpretations against the raw data, and conduct an external check on the study process to boost credibility. The study sites for the findings’ transmission were unknown to the principal investigator and researchers. All research processes were logical, traceable, and documented to ensure dependability. Furthermore, in this study, the research’s interpretations and conclusions relied heavily on the collected data as a reliable source. To ensure the dependability, credibility, transferability, and confirmability of the findings, all necessary measures were implemented in the research^(42)^ (see Table 1).

**Table 1.** The four-dimensions criteria (credibility, dependability, confirmability, and transferability) strategies adapted from Lincoln and Guba^(42,43)^

Rigour criteria	Purpose	Original strategies	Strategies applied in our study to achieve rigor
Credibility	To establish confidence that the results (from the perspective of the participants) are true, credible and believable	• Interviewing process and techniques	• Interview protocol was tested before application and pilot interviews were conducted
		• Establishing investigators’ authority	• We ensured the investigators had the required knowledge and research skills to perform their roles
		• Collection of referential adequacy materials	• We asked interviewers to send all the field notes to the principal investigator for analysis and storage.
		• Peer debriefing	• We had regular debriefing sessions with multi-disciplinary co-authors
Dependability	To ensure the findings of this qualitative inquiry is repeatable if the inquiry occurred within the same cohort of participants, coders, and context.	• Rich description of the study methods	• We prepared detailed drafts of the study protocol throughout the study.
		• Establishing an audit trail	• We developed a detailed track record of the data collection process. Keeping records of the raw data, field notes, transcripts
		• Stepwise replication of the data	• We measured the coding accuracy and inter-coders’ reliability of the research team.
			• ensure the research process is logical, traceable, and documented
Confirmability	To extend the confidence that the results would be confirmed or corroborated by other researchers	• Reflexivity	• Periodic investigators and coauthors meetings.
		• Triangulation	• We applied several triangulation techniques (methodological, data source, investigators, and theoretical).
		• Establishing that the researcher’s interpretations and findings	• Reasons for theoretical, methodological, and analytical choices throughout the entire study, so that others can understand how and why decisions were made.
Transferability	To extend the degree to which the results can be generalised or transferred to other contexts or settings	• Purposeful sampling to form a nominated sample	• We used a combination of three purposive sampling techniques.
		• Data saturation	• We quantified operational and theoretical data saturation.

### Study procedure

In this study, we used and applied Lincoln and Guba’s established strict standards known as credibility, dependability, confirmability, and transferability for determining and enhancing the trustworthiness of the research. To assess and ensure the robustness of the study, we carefully organized and carried out a series of semi-structured interviews and FGDs based on the above-mentioned criteria. These standards have been applied in numerous qualitative studies on health in the past.^(44)^

### Ethical considerations

The research study obtained ethical approval (Ref. No. CNCSDO/623/15/2023) from the CNS-IRB of Addis Ababa University, aligning with the Declaration of Helsinki. Ethical practices with student participants included using age-appropriate consent forms, obtaining parental permission and student assent, ensuring confidentiality, emphasising voluntary participation, and obtaining ethical approval. Participants were informed of their right to withdraw without consequences. Written consent and assent were obtained from all participants, adhering to confidentiality requirements, and all individuals participated voluntarily. Permission was granted by the Addis Ababa Education Bureau.

## Results

### Socio-demographic characteristics

The study included a diverse range of participants: mothers (aged 25–45), school directors (aged 25–52), and students (aged 12–19). Interviews were conducted with 20 students, 20 school administrators, 3 Ministry of Education experts, 4 sub-city experts, and 3 School Feeding Agency experts. FGDs involved 48 mothers of beneficiary children, with an average of 10 participants per group (6–12 mothers per group). In total, the study had 98 participants and aimed to examine the benefits and challenges of homegrown SFPs in Addis Ababa. See Table 2 for more information.

**Table 2.** Socio-demographic characteristics of study participants

Participants	Gender	Age range	Number of study participants
Mother	Female	25–45	48
School director	Mixed (F/M)	25–52	20
Students	Mixed (F/M)	12–19	20
Ministry of education expert	Mixed (F/M)	35–55	3
Sub-cities experts	Mixed (F/M)	30–42	4
School feeding agency experts	Mixed (F/M)	32–46	3
Total study participants			98

### Themes and sub-themes

Although there have been many challenges to the implementation of homegrown SFPs in Addis Ababa, this study only identified two main themes and twelve sub-themes. Perceived benefits of SFPs and perceived challenges to homegrown SFPs emerged as the two main themes. For a detailed description of the themes and subthemes, please refer to Table 3.

**Table 3.** Summary of themes and their respective sub-themes

	Themes		Sub-themes
1	Perceived benefits of school feeding program	1	Improved academic performance, class attendance, attention and, and reduced dropout rates and class repetition
		2	Reduces the socioeconomic burden of the family
		3	Improved student behaviour and reduced disruptive behaviour
		4	Reducing psychosocial stress and increasing social integrity
2	Perceived barriers and challenges to homegrown school feeding program	5	Underpayment of workers
		6	The poor market linkage between fostering mothers and consumer cooperatives
		7	Poor infrastructure
		8	Increased sense of dependency
		9	Increase workload for school staff
		10	Provision of poor-quality food
		11	Lack of adequate collaboration between the government and stakeholders
		12	Lack of linkage between SFP and school gardening

### Theme 1: perceived benefits of the home grown school feeding program

The significance of the HGSFPs in preventing hunger among students from low-income households was attested to by all FGD participants, students, and key informants. Additionally, the mothers of the students have acknowledged this. According to the key informants from schools, the program has helped to cut dropout and class repetition, and improved school attendance, academic performance, and concentration of students, Reportedly, before the initiation of the program, some students used to come to schools without lunch boxes and sometimes students collapsed in classes due to hunger. Arriving late for class, which used to be a big problem for the schools, has been solved since the program started. The perceived benefits of SFPs in Addis Ababa, Ethiopia, were found to be categorized under the following themes.

#### Sub theme 1.1: improvements in student attendance, concentration, academic performance, school dropout, and class repetition reduction

Most of the participants of this study explained that the school feeding program has improved students’ attendance, academic performance, and completion through reduced hunger. They also believed that providing school meals incentivizes households to send their children to school through a transfer (the daily meal) that is intended to help offset the financial and opportunity costs of schooling.

One of the key informants explained that before the provision of school meals, students who arrived at school on an empty stomach had trouble focusing on their education. Now, school meals can provide immediate relief from hunger, reducing distraction and increasing attention span among students. One of the FGD participants explained that the program also prevents students from missing school due to hunger, which saves parents time and money by reducing the amount of meal preparation required.

*“Imagine the severity of the issue that caused mothers to send their kids to school with empty lunch boxes……I observed students carrying empty lunch boxes even before the program started.”* SFP monitoring expert, sub-city education office

*“Previously, we used to conduct action research to reduce students late coming to school. Yet, there is no longer any worry following the SFP’s implementation.”* SFP improvement and monitoring team leader, sub-city education office

*“Most of the time go to school with an empty stomach, so I feel tired and sleepy. However, since the feeding program started in our school, I get food every day, and feel energetic; I don’t feel hungry anymore. Now I am happy and enjoy the class”* (An interview from an SFP beneficiary student)

*“Had poor academic performance and no interest in learning before the school feeding program, but now he is much more interested in learning and his academic performance has considerably improved.”* (An interview from SFP beneficiary student)

#### Sub theme 1.2: reducing burdens on the family

In addition to positive effects on education, the school feeding program offers several socio-psychological benefits. According to FGD participants, parents are relieved from the financial and physical strain of cooking meals for their school-age children every day. Additionally, this has indirectly increased food security in the home through the phenomenon known as spillover effects, in which the family’s usage of one resource by one child may help another. Children from poor households are no longer suffering from the psychological pressure of being unable to bring lunch to school.

The program has also made kids happy and focused. The majority of participants concurred that the school feeding program lessened the financial strain on students’ families as well as the stress placed on them associated with their poor economic status. One of the FGD participants said the following “I work at this elementary school, where my daughter and son attend. Because of my low monthly income, I find it difficult to feed my entire family. Before the feeding program, I was unable to give my school-age children breakfast and lunch, but now, thanks to the government, the problem has been rectified.”

*“I had a lot of difficulty deciding what my children should eat for breakfast and lunch before the school feeding program started, as well as what I should cook for them. Most of the time, they went to school without eating breakfast or holding their lunch. Now, thanks to God, my stress has been resolved.”* (FGDs participants).

In Addis Ababa, the homegrown school feeding program has so far only covered elementary schools, and the program has not reached high school students. As reported by sub-city education offices, poor students get challenged as they advance to high school. Many high school students from economically disadvantaged families stay the whole day at school without lunch boxes, while others share the lunch with other students.

*“High schools have to benefit from the program as well. After entering high schools, underprivileged students who were previously served by the program are having difficulty, and some high school students have illegally entered the nearby elementary school to get school meals.”* (A school director, at a primary school)

#### Sub theme 1.3: change in students’ behaviour

The majority of key informants believed that students had improved behaviour and safety at school as a result of the school feeding program. Besides, respondents said it increased the student’s motivation to avoid harm, go to unnecessary places, and skip school compounds. Before the implementation of HGSFP, students reported used to climb over the school fence to look for food when they became hungry. Additionally, they explained that when students go looking for food, they get exposed to crime or threats, sexual abuse, harm, or harassment, and get victimized. Student violence is supported and influenced by out-of-school gangs, juvenile delinquents, street drug dealers, and drug addicts.

According to one of the key informant explanations, before the implementation of the homegrown school feeding program, many students were subjected to unnecessary behaviours such as chewing khat and shisha. Since the introduction of the school meal program, they have stayed at the school the whole day, and as a result, they are safer than ever and focusing only on their education.

*“Various misconduct offenses, such as those that disrupt the learning environment and those that involve aggressive behavior, such as fighting, bullying, and student assault, were observed on students before the implementation of the school feeding program, but these disciplinary issues have now significantly decreased.”* (A school director, at a primary school)

The majority of participants in focus groups and key informant interviews acknowledged that food insecurity and hunger among children have a detrimental impact on their academic focus, performance, and behavioural patterns. Moreover, the majority of research participants reported that the HGSFP has led to a reduction in student misconduct, including conflicts with school security guards, class skipping, and financial theft from parents. Notably, access to free school meals, especially, has been associated with a decrease in disciplinary infractions.

*“Before the school feeding program started, some students used to steal money from their parents to buy bread and various sweets and spend the stolen money. It was causing them to grow up with inappropriate behavior, but after the school feeding program was started, the problem was significantly reduced.”* (FGD participants)

*“Before the implementation of the school food program, there were numerous behavioral issues among students, and occasionally, students would fight with the school security staff for various reasons. However, now these issues have significantly decreased.”* (A school director, at a primary school)

#### Sub theme 1.4: decreased psychological stress and increased social integrity

Before the start of the school feeding program, only students from low-income families were eligible for the program, which subjected students to discrimination, as was commonly mentioned by some FGD and key informant participants. Furthermore, the majority of participants also described that the SFP increased unity and social integrity and decreased discrimination among students when they ate together, regardless of their families’ economic status.

*“Students are protected from psychological harm and shame by the school meal program. Many students did not bring food to school prior to the school feeding program’s implementation, and those from families with comparatively higher incomes brought lunch boxes. This caused psychological trauma for the students, but these issues have since been resolved thanks to the government, and all students now share the same meal*.*”* (A school director, at a primary school)

*“The school feeding program helps alleviate perceived discrimination, instead fosters strong relationships with students through eating together and enabling them to concentrate on their studies by providing equal access to services for all students.”* (FGDs participant)

### Theme 2: perceived challenges to the HGSFP in Addis Ababa city

The Addis Ababa City Administration School Feeding Agency recently assigned 1-2 nutrition experts to each school, which improved program quality and encouraged ownership. The experts oversee the program’s execution and offer technical support. Although assigning nutrition experts at the school level has been taken as a positive step, sub-city and district education offices continue to lack the staff needed to consistently offer schools supportive supervision.

The women groups receive 20 birr per student payment to prepare two meals per day for each student. With this modest budget and given the ongoing food price inflation, the women are struggling to prepare the meals according to the standard menu. Due to budget shortages, the women are losing interest, and sometimes they provide substandard meals.

#### Sub theme 2.1: underpayment of workers

The majority of key informant interviews and focus group discussion participants agreed that the salary currently provided to women who prepare meals for students is insufficient to cover inflation or the actual cost of living. During the focus group discussion, a study participant mentioned that food handlers face a lot of pressure due to their low salaries, which makes them demoralized at work and forces them to take on other jobs like housecleaning and washing clothes to support their families. As a result, there might be a detrimental effect on the quality of services provided. According to one of the key informants, the government must assess the food handlers’ payment, and before determining how much to pay for them, a market evaluation study that takes into account the challenges imposed by the current cost of living must be conducted.

According to one of the key informant’s opinions, with the existing budget, the quality of school meals may deteriorate to the extent that it threatens the very significance of the program. The current food price inflation is even pushing HGSFPs run by NGOs to compromise their meal plans.

*“A daily budget of 20 birr is set aside for breakfast and lunch for each student. It is challenging to feed children on this budget in the current market.”* SFP monitoring expert, sub-city education office

*“An egg currently costs 12 or 13 birrs. So, how can you feed a student for two meals with a budget of 20 birr a day?”* SFP improvement and monitoring team leader, sub-city education office

*“A kilogram of bananas cost 25 birr when our school feeding program first began; the price has now doubled. Thus, the banana must be eliminated from the menu.”* Respondents from an NGO implementing SFP

The majority of FGD participants explained that they were unhappy with the refusal of the government not to pay for food handlers during maternity leave and the summer when schools are closed. Food handlers lack alternate means of income, which makes it difficult for them to support their families and lead during this time as a result of these issues.

*“The problem of not paying the mothers who prepare food for the students after June 30 when the school is closed. Along with this, breadwinner mothers are exposed to problems as they will not have monthly income until the school opens.”* (FGD participant)

*“During maternity leave, the government is not paid for food handlers like other workers, and due to this feel discrimination and dissatisfied with their work.”* (FGD participant)

#### Sub theme 2.2: the poor market linkage between fostering mothers and producers

The major challenge explained by FGD participants and key informant participants was the lack of market connections or linkage between foster mothers and suppliers of market items and agricultural products. Some participants indicated that the HGSFP’s inability to obtain essential supplies like tef, flour, oil, sugar, and bread sufficiently, as vegetables and fruits did not get.

The women groups providing school meal service have some market linkage with consumer cooperatives (specifically for sugar, flour, and oil) and Sheger Bakery. However, according to the key informants, the linkage is not adequate to financially sustain the program. Schools also do not have any established linkage for other supplies like vegetables and fruits. As reported by the key informants, the cost of grains and vegetables might have been lower if there had been direct market connections established with farmers or agricultural cooperatives. Despite the government’s previous promises, there has been no development of this type of market linkage.

Thus, women’s unions that organize food for public school children in Addis Ababa face a threat to their sustainability due to the current inflation and rising prices of supplies and commodities in the city. The mothers who participated in the FGDs explained that they were unhappy working as cooks in the school feeding program because the payment they received did not consider the high workload and the current market inflation.

*“There is a link between sugar, oil, flour, and the sheger bread association, but it is insufficient because it does not account for the quantity and number of students.”* (FGDs participant)

#### Sub theme 2.3: poor infrastructure

Most participants in focus groups and key informants agreed that schools did not have the necessary infrastructure for a successful school food program. Many schools also do not have adequate dining halls to accommodate students; therefore, classrooms and libraries have to be used for the same purpose. Also, there is a lack of standard kitchens and standard stores; instead, they use the floor or improvised areas for food storage. As a result, the school feeding program’s food safety is at risk due to the aforementioned issues.

Even though every school in the city has access to safe drinking water, the program has been impacted by the regular outages of water supplies and electricity, as well as the absence of conventional water storage facilities. Since most schools currently lack access to three-phase electricity, which is necessary for mass cooking, biomass fuel is used instead.

*“Among the public primary schools under the Addis Ababa City Administration, only a few have standard kitchens. It is difficult to talk about food safety issues while the kitchen is substandard”* SFP improvement and monitoring team leader, sub-city education office

*“Frequent water and electricity outages in our schools had a major challenge on the implementation of the school feeding program.”* (A school director, at primary schools)

*“Most schools’ kitchens for preparing meals for students are made of tin, which prevents sufficient airflow. So it is very difficult for the mothers to do their work and risk for their health.”* (FGDs participant)

#### Sub theme 2.4: increased sense of dependency

One of the school directors explained during a key informant interview that the school feeding program causes families of children to feel dependent on the program. Another school director stated during a key informant interview that the school feeding program forces students to refocus their attention on school meals rather than their schoolwork and causes them to spend too much time eating at the nearby dining room, not providing enough time for studying.

*“School feeding programs are beneficial for students. However, it would be preferable if it just applied to low-income parents of students. The school feeding program has unnecessarily raised dependence on the parents of students. They are no longer able to take on responsibility or handle problems alone, and as a result, they develop a dependency mentality.”* (A school director, at primary schools)

#### Sub theme 2.5: increased work burden

The initiation of the HGSFP has also caused a burden for the school in terms of managing operational issues and facilitating the finances needed for the program. The HGSFP has increased the work burden on principals, teachers, and admin staff. The feeding program is also compromising the time allotted for learning activities. However, considering the benefits of the program, the school community has so far taken on the burden of positivity.

*“SFP has increased the work burden on school directors, teachers, and administrative staff. But they are aware of the benefit of the program as well.”* SFP improvement and monitoring team leader, sub-city education office

*“Some students give a lower priority to their educational activities, including study time, rather than spending much time eating breakfast and lunch.”* (A school director, at primary schools).

#### Sub theme 2.6: provide poor meal quality

The Addis Ababa School Feeding Agency (SFA) prepared the current school menu to diversify diets, standardize meals, and ensure that children’s nutrient needs are satisfied. The SFA has introduced standardized recipes based on locally available ingredients.

Despite adhering to a set menu, some key informants have expressed concerns about the nutritional value of school meals. Financial constraints prevent the inclusion of animal-based products like milk and eggs, while limited resources make it challenging to provide sufficient fruits and vegetables. As a result, the quality of the meals served to students may be compromised. Key informants from sub-city education offices have reported that bulk preparation sometimes leads to unappealing school lunches, affecting student satisfaction.

During a key informant interview, one of the school directors indicated that the lack of adequate funding for the school feeding program made it impossible to include animal products, fruits, and vegetables in the food menus. A student who participated in this study expressed dissatisfaction with the current food quantity and quality provided in the school feeding program. Another student also provided the following explanation: “We are unhappy because the food they received throughout the week was not varied and had no animal products, like eggs, milk, and meat.”

*“Sometimes students bring lunch boxes from home complaining that the school meals are not palatable”* SFP improvement and monitoring team leader, sub-city education office

#### Sub theme 2.7: engaging diverse stakeholders in HGSFP

According to a key informant from the Ministry of Education (MoE), with the recent food price inflation, it is not possible to fully finance SFP with the government budget alone. So, efforts have to be made to mobilize resources from the community and other partners. There is also increasing interest from NGOs to support the HGSFP. However, the collaboration between the School Feeding Agency (SFA) and NGOs engaged in HGSFPs is far from ideal. Effort to engage individual local contributors is also low.

Although the Addis Ababa City Administration greatly needs multi-stakeholder engagement, there is currently no clear government directive on how to involve NGOs in school food programs. Instead, the School Feeding Agency is pressuring NGOs and contracting out the food preparation to a local women’s union, which has lowered the standard of services offered to students.

#### Sub theme 2.8: linkage between HGSFP and school garden

According to a key informant from the Ministry of Education (MoE), some schools in the Amhara, Oromia, and SNNP regions have started school gardening to strengthen their HGSFP. The respective regional structures of the agriculture sector are supporting the initiative by providing technical and material support, including agricultural inputs like improved seeds. Experience from elsewhere outside Addis Ababa indicated that school gardening has improved access for schools to a fresh supply of vegetables and fruits. Further, it has also excluded middlemen from the market chain and reduced the expenses of the program.

*“One major approach to ensuring students access to fresh and nutritious vegetables and fruits is the initiation of school gardens.”* Health and Nutrition Expert, MoE

In contrast, the achievement of school gardening is modest in Addis Ababa because, unlike rural schools, schools in the city do not have adequate space to initiate gardening at scale. The Addis Ababa City Administration (AACA) still has not initiated urban agriculture within the school environment. High schools in Addis Ababa are comparatively bigger than elementary schools. However, since high schools are not currently enrolled in the HGSFPs, this cannot be directly linked to the HGSFP.

School gardening has the potential to improve the accessibility of the SFP and to get vegetables at better prices and quality. This has also been observed in schools that started gardening. So far, most schools that run SFP in Addis Ababa have not started gardening. Many of the schools have no adequate space to establish meaningful urban agriculture, while others have not received technical support for doing so. Those had experience with school gardening, scarcity of water, lack of personnel to take care of the farm, and limited production. Many key informants assumed urban agriculture may help students understand how agriculture works. Furthermore, it will serve as a demonstration site for the agriculture course that will be included in formal education starting in the coming year.

One major challenge that hinders the linkage between urban agriculture and SFP is the low productivity of school gardens. Experience from the schools that implemented urban agriculture at different levels suggests that the scale of production is too small to have a meaningful effect on the meals delivered to students.

*“The existing space in schools does not allow for large-scale production. However, school gardening is used as a demonstration center so that students will apply the experience at home.”* SFP monitoring expert, sub-city education department

*“We are implementing school gardens and we have been acknowledged for that. However, the demand of the HGSFP is large and cannot be met by the gardens alone.”* A SFP coordinator

## Discussion

Our study aimed to address gaps and limitations in previous research on HGSFP. To fill these gaps, we conducted a qualitative phenomenological study in Addis Ababa, Ethiopia, comprehensively exploring the perceived benefits and challenges of HGSFP. Our findings indicate improved student nutrition and education outcomes but persistent challenges in infrastructure, logistics, operational, and financial issues. These findings align with previous research emphasising the importance of contextual factors in HGSFP implementation.

In terms of benefits, our study identified increments in school enrollment, attention span, academic performance, and attendance, along with reductions in dropout rates and class repetition. The program also alleviated the socio-economic burden on students’ families, decreased social psychological pressure, improved social integrity among students, and prompted changes in students’ behaviour. However, challenges such as poor-quality meal provision, lack of market linkage, poor infrastructure, underpayment of workers, dependency on the program, lack of collaboration between stakeholders, and inadequate linkage between HGSFPs and school gardening were identified as key barriers in the effective and efficient implementation of HGSFPs in Addis Ababa City, Ethiopia.

Our research enhances the theoretical understanding of homegrown school feeding programs (HGSFP) by revealing the intricate interplay between local contexts and program effectiveness. These findings can serve as a foundation for future studies to explore long-term impacts and formulate scaling strategies specifically for urban environments. Offering school meals has multiple benefits, including improved academic performance, increased enrollment, attendance, retention, and completion rates for pre-primary and primary school students. It also reduces hunger, enhances students’ health and nutritional status, and helps break the generational cycle of malnutrition. Additionally, providing daily meals lowers the opportunity and financial costs of sending children to school, encouraging families to prioritize their education.

According to the results of the current study, The feeding program has improved enrollments, decreased the dropout rate, improved academic achievement and concentration, and decreased absenteeism and dropout rates. Thus, the HG-SFPs may have enhanced educational and nutritional outcomes by reducing short-term hunger. This keeps students from having to leave school to get food and makes them more interested in class. This result is consistent with a quantitative study conducted in Addis Ababa, Ethiopia,^(14,45)^ which revealed a favorable relationship between academic achievement and SFP.

This result is in line with a review^(46)^ of qualitative studies that examined the consequence of SFP on the education outcomes of students’ such as enrollment, completion, and academic success and were conducted in Nigeria and Niger.^(47,48)^ Therefore, strengthening the SFP could be a crucial intervention to enhance students’ academic performance and improve the quality of their education.

The lessened socioeconomic strain on student families was the other perceived advantage of HGSFPs in Addis Ababa. It was difficult for low-income families to feed their kids breakfast and lunch, and when students went hungry and looked for food, the families experienced worry and anxiety. However, the HGSFPs helped them reduce the pressure that society’s socioeconomic system placed on families, especially those with lower incomes.

This finding is supported by a systematic review report,^(46)^ and a study conducted in the Sidama region of Ethiopia.^(14)^ This conclusion was further supported by a Tennessee, USA study that found school lunch programs decreased family stress regarding time and money spent on food shopping, cooking, and packing for their kids.^(49)^ Therefore, the Homegrown School Feeding Program (HGSFP) has the potential to be a crucial strategy for enhancing the quality of life for families, particularly those with low incomes.

According to the study, the School Feeding Program (SFP) led to a reduction in social psychological pressure and an increase in social integrity among students. Previously, there was a school feeding program exclusively for students from the poorest families, which placed psychological pressure on the students. However, with the endorsement of the Homegrown School Feeding Program (HGSFP) in all public primary schools in Addis Ababa, this pressure has been alleviated.

Currently, there are no eligibility requirements for students to receive breakfast and lunch at school, irrespective of their parents’ financial situation. This inclusive approach has led to a decrease in psychological stress and an enhancement of social integrity among students. This finding is supported by evidence from a qualitative study conducted in India^(50)^ and a systematic review that reported an increase in social interaction and integrity among students through the School Feeding Program (SFP).^(46)^

The HGSFPs contribute to reducing poverty both directly and indirectly through improving community wellbeing.^(47)^ The study found that implementing the Homegrown School Feeding Program (HGSFP) resulted in a decrease in inappropriate student behaviours, including class absenteeism, substance use, and exploitation. This finding is consistent with a study conducted in Niger, which also highlighted the role of SFPs in reducing student misconduct such as theft, drug use, sexual assault, and delinquency.^(47)^ This suggests that HGSFP could be applied as a strategy for generating a disciplined and productive generation in the future.

Ethiopian school feeding policy made clear direction that the government is responsible for providing schools with basic facilities that are appropriate for the local environment.^(19)^ However, this study indicated that inadequate facilities, such as those in the kitchen, food storage, dining room, water supply, and electric supply, were among the main obstacles to the implementation of HGSFP. When there was no water in the school, the food handlers paid laborers to fetch water, and when the electricity went out, the food handlers used a wood fire to cook the food instead, which exposed them to hazardous smoke. Qualitative research in the Sidama region of Ethiopia,^(14)^ Nigeria,^(48)^ and Uganda^(51)^ as well as a qualitative systematic review undertaken globally,^(46)^ all provide credence to this finding. Therefore, building basic facilities as well as improving the water and electric supply could positively impact the success and sustainability of HGSFPs in settings with scarce resources, such as Ethiopia.

This study also indicated that underpayment of workers under HGSFP, absence of payment for workers in the summer season, maternity leave, and budget that doesn’t consider inflation were other challenges to the effective implementation of HGSFPs. This finding is supported by evidence from a qualitative systematic review in a global context^(46)^ and a qualitative study conducted in Addis Ababa.^(52)^ These studies reported that the absence of a budget that considers inflation was a challenge for the smooth implementation of HGSFPs. Therefore, this program should give due emphasis on adjusting the budget according to the living cost of the country.

In this study, a key obstacle to effective Homegrown School Feeding Programs (HGSFPs) was the lack of networking among producers, suppliers, and the program. This resulted in shortages of essential supplies and commodities for the HGSFP, a finding supported by a global review of SFPs.^(46)^ Thus, it is crucial to prioritize the improvement of the food supply chain, as emphasized in the school feeding policy.^(19)^

According to Ethiopian school feeding policy,^(19)^ effective and sustainable HGSFPs need cross-sectoral cooperation, and the local community is expected to oversee the implementation and allocation of resources with a sense of ownership. The HGSFPs were, however, found to be developing a sense of dependency among the parents of the students. Thus, to maximize the sense of ownership and minimize the sense of dependency on it, as already mentioned in the national school feeding policy, program implementers should pay appropriate attention to engaging parents of students in particular and the local community in general in the process of planning, implementing, and evaluating this program.^(19)^

### Strengths and limitations

One of the strengths of this study is the inclusion of diversified potential study participants who have in-depth information and rich insight into the homegrown school feeding program. Furthermore, the interview facilitators were proficient in the local language, well-versed in the community, and had prior experience with qualitative research. Relationships were established before data collection by approaching the primary school directors for prior permission. The analysis was conducted as a team with the help of multiple researchers. Considering the study’s limitations, its qualitative nature limits the generalisation of its findings due to the lack of a representative sample. This study would have benefited more from a mixed-methods approach to quantifying the size of each perceived benefit and challenge associated with HGSFPs so that planners and policymakers may more easily prioritize problems.

## Conclusions and recommendations

Awareness-raising efforts should be started to address the parent’s incorrect perception of their dependence on the school feeding program. To lessen problems and enhance the quality of HGSFP’s, all primary schools must fulfill the necessary infrastructure for the program, like constructing standard dining halls that can accommodate the number of students as well as standard food storage and cooking areas. They also must have ready-to-use water storage facilities and private generators.

In addition, the government should establish market links for fruits and vegetables, which will contribute to the HGSFPs and meet the nutritional needs of the students. Furthermore, starting and supporting urban agriculture and school gardening should be the alternative strategy. Besides, direct market connections with agricultural cooperatives are essential for decreasing the current inflation of food prices and securing food supplies at lower costs.

To improve the efficiency of the HGSFP, lessen the workload on administrators, teachers, and other staff members, and streamline the handling of program-related operational and financial issues. It is better to entirely outsource to the appropriate body. In addition, it is important to assign the right people to each position, clearly define roles and responsibilities, and allocate funds at all levels.

According to the researchers, future studies should be needed, to quantify the perceived benefits and drawbacks of HGSFP to make it straightforward for policymakers and program designers to prioritize problems based on their magnitude. The HGSFPs are not stand-alone interventions; therefore, they need to be strengthening the network of local private and public partners and NGOs working in HGSFP-related fields by developing a coordinated partnership strategy with well-defined roles, management, financial responsibilities, and implementation guidelines.

In order to overcome the operational and strategic obstacles to the program and improve the effectiveness of the domestic school feeding program, a framework for coordination between the various sector ministries and task forces must be established.

## Supporting information

Tamiru et al. supplementary material 1 Tamiru et al. supplementary material

Tamiru et al. supplementary material 2 Tamiru et al. supplementary material

Tamiru et al. supplementary material 3 Tamiru et al. supplementary material

Tamiru et al. supplementary material 4 Tamiru et al. supplementary material

Tamiru et al. supplementary material 5 Tamiru et al. supplementary material

Tamiru et al. supplementary material 6 Tamiru et al. supplementary material

Tamiru et al. supplementary material 7 Tamiru et al. supplementary material

Tamiru et al. supplementary material 8 Tamiru et al. supplementary material

Tamiru et al. supplementary material 9 Tamiru et al. supplementary material

## Abbreviations:

**FGDs:** Focus group discussions; **HGSFP:** Home-Grown School Feeding Program; **KII:** Key informant interview

## Supplementary material

The supplementary material for this article can be found at https://doi.org/10.1017/jns.2024.42
