# Supplementary material for: Perceived benefits and challenges of school feeding program in Addis Ababa, Ethiopia: a qualitative study
Source: J Nutr Sci. 2024 Sep 18;13:e32. doi: 10.1017/jns.2024.42 (PMC11418071; doi:10.1017/jns.2024.42)
Supplement: Tamiru et al. supplementary material 1 — Tamiru et al. supplementary material [file S2048679024000429sup001.docx]

**FGD Discussion Guide: Beneficiary Students' Mothers**

Thank you for joining us today. I appreciate your time and willingness to participate. My name is ___, and we would like to discuss your opinions and experiences regarding the homegrown school feeding program in Addis Ababa. Our aim is to understand the challenges and perceived benefits of the program so that we can improve future interventions. The interview will take approximately an hour, and I will be recording the session to ensure accuracy. While I will be taking notes, please speak clearly to ensure that your comments are captured. Rest assured, all your responses will remain confidential. They will only be shared with our research team, and any information included in our report will not identify you as the respondent. Please remember that you are not obligated to discuss anything you are uncomfortable with, and you may end the interview at any time. Do you have any questions or clarifications before we proceed? Are you willing to participate in this interview?

Thank you once again for your participation.

**Interview Guide for FGD Participants/Parents of Beneficiary Students**

**Demographic information:**

1. School ………………
2. Age ………….
3. Gender …………..
4. Highest qualification?. Certificate ( ), Diploma ( ), Degree ( ),Master’s degree ( )
5. Occupation………………..

**Perceived benefits of HGSFPs**

1. As a parent or guardian, how do you feel about the homegrown school feeding program?
2. Do you believe it is appropriate to have the Homegrown School Feeding Program in schools? If yes, why do you think so?
3. What benefits do you think the program has brought to you and your children?
4. Tell me about the socio-economic challenges faced before the implementation of the homegrown school feeding program
5. Have you noticed any changes in your family's income after the implementation of the home grown school feeding program? If yes, could you explain?
6. How does the Homegrown School Feeding Program reduce the socio-economic burden on families?
7. What benefits do you think the program has brought to the welfare of your child?
8. Do you believe the Homegrown School Feeding Program influences students' academic performance in schools? If yes, please provide reasons for your answer.
9. What are your overall views on the Homegrown School Feeding Program in schools?
10. Do you think it is appropriate to have the Homegrown School Feeding Program in schools? If yes, why do you think so?
11. According to your opinion, what could be the challenges faced by the school administrators as regards the homegrown school feeding program?
12. What do you believe should be done to improve the Homegrown School Feeding Programs in Addis Ababa City?

Thank You!
