# Supplementary figures and images for: Perceived benefits and challenges of school feeding program in Addis Ababa, Ethiopia: a qualitative study
Source: J Nutr Sci. 2024 Sep 18;13:e32. doi: 10.1017/jns.2024.42 (PMC11418071; doi:10.1017/jns.2024.42)

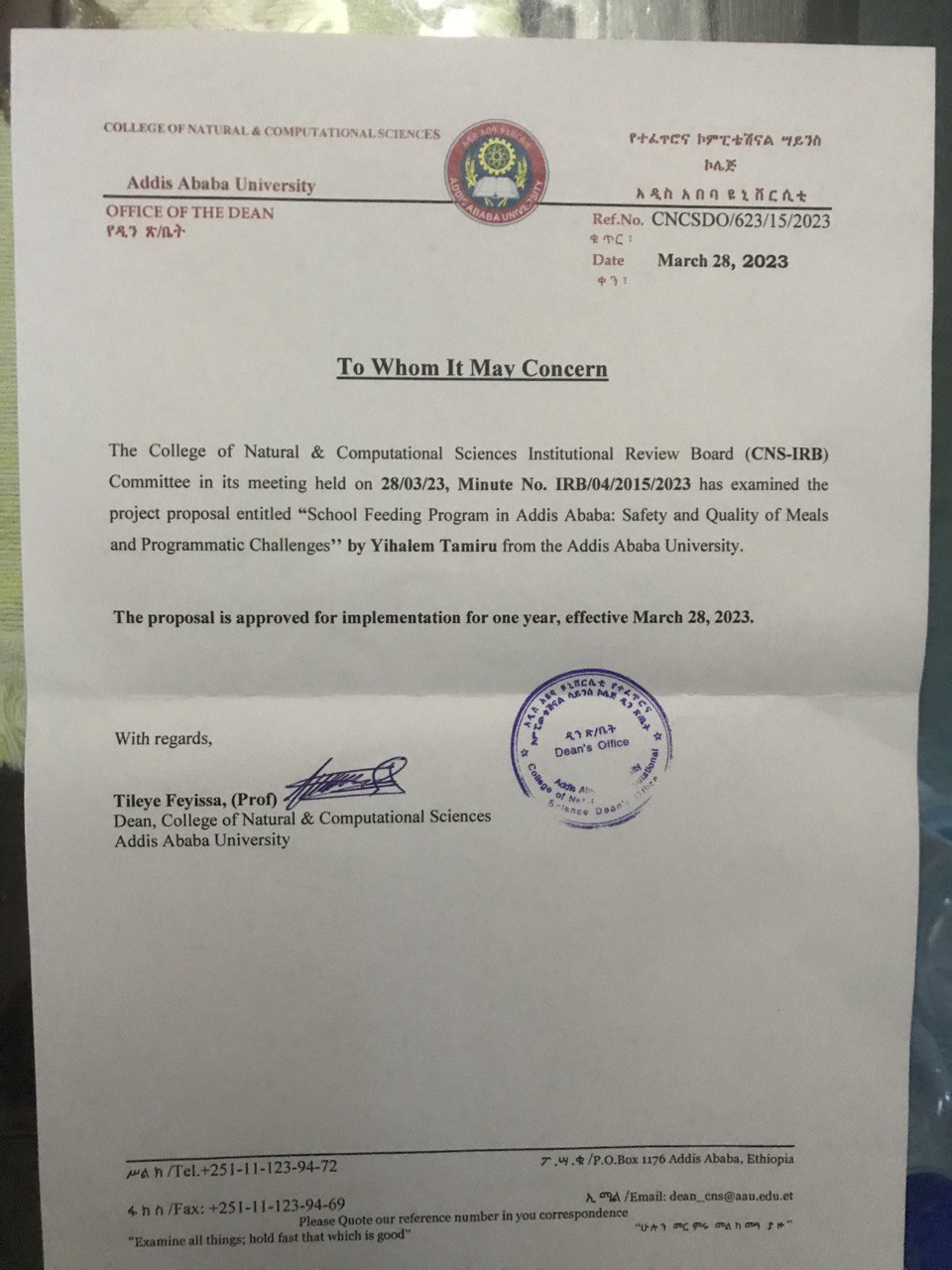

Supplement: Tamiru et al. supplementary material 2 — Tamiru et al. supplementary material [file S2048679024000429sup002.zip › IRB LETTER photo_2023-10-04_16-16-32.jpg]
