# Supplementary material for: Perceived benefits and challenges of school feeding program in Addis Ababa, Ethiopia: a qualitative study
Source: J Nutr Sci. 2024 Sep 18;13:e32. doi: 10.1017/jns.2024.42 (PMC11418071; doi:10.1017/jns.2024.42)
Supplement: Tamiru et al. supplementary material 3 — Tamiru et al. supplementary material [file S2048679024000429sup003.docx]

**Informed Consent for FGDs participants**

Dear Respondents,

I want to thank you for taking the time to meet with me today. My name is ____, and I would like to talk to you about your opinion and experience with the homegrown school feeding program. Specifically, the challenges and perceived benefits of a homegrown school feeding program in Addis Ababa We are assessing the perceived benefits and programmatic challenges of homegrown school feeding programs.

I believe there are no risks to you from participating in this study. Taking part in this research study may not benefit you personally, but it may help us improve future school feeding program interventions while at the same time improving the nutritional, health, and educational outcomes of primary school students. You will not be compensated for taking part in this study. I appreciate your willingness to take part in this research.

If you agree to participate in this study, I would like to question you about your opinion, view, and understanding of the perceived benefits and challenges of a homegrown school feeding program in Addis Ababa, Ethiopia. This study will help with future improvements to the school feeding program. The FGDs might take 60 to 90 minutes to finish. Taking part in this research study may not benefit you personally, but it may help the government improve the program and, at the same time, improve the educational and nutritional outcomes of primary school students. I believe there are no risks to you from participating in this study, and you will not be compensated for taking part in it. I appreciate your willingness to take part in this research.

To the best of our ability, we will keep your responses private. When we present this study to others or publish its findings, your name and other identifying information will be removed. Any information that may be used to identify you will be kept separate from your responses, and your responses will be held in a safe location for research purposes only. The data will be saved on password-protected computers.

It is voluntary to take part in this study. There are different points of view, rather than right or wrong answers. Do not hesitate to express your opinions, even if they diverge from those of others. Remember that we value critical comments equally to good ones and that sometimes the latter is the most insightful. We're capturing the session on tape, so we won't miss any of your feedback. In these talks, people frequently make insightful comments, and we are unable to record them all promptly. No names will appear in our reports. You have my word that everything will remain private.

Remember, you don’t have to talk about anything you don’t want to, and you may end the interview at any time. Are there any questions about what I have just explained? If you have any questions, comments, or complaints, you can reach the researchers through the following addresses: Tel. +251911378982, email: yihalemtamiru@gmail.com, or his supervisor, Dr. Samson Gebremedhin, Tel. +25191682281, e-mail: samsongmgs@yahoo.com.

The Institutional Review Board of Addis Ababa University has reviewed this project. For any concerns regarding this study or if you feel that your rights as a participant (or the rights of another participant) have been violated or have caused you undue distress (physical or emotional distress), please contact the IRB secretary, Dr. Aynadis, by phone during normal business hours at +251912062604 or [irb.cncs@aau.edu.et](mailto:irb.cncs@aau.edu.et).

I have read and understood the above information. I give consent to participate in the current study.

Name of the FGD participants for whom consent is asked: _______      Signature:__________

Signature of the data collector: ___________     Date:______________

Signature of Investigator:______________ Date: ______________
