# Supplementary material for: Perceived benefits and challenges of school feeding program in Addis Ababa, Ethiopia: a qualitative study
Source: J Nutr Sci. 2024 Sep 18;13:e32. doi: 10.1017/jns.2024.42 (PMC11418071; doi:10.1017/jns.2024.42)
Supplement: Tamiru et al. supplementary material 4 — Tamiru et al. supplementary material [file S2048679024000429sup004.docx]

**Informed Consent for key informant interview**

Dear Respondents,

I want to thank you for taking the time to meet with me today. My name is ____, and I would like to talk to you about your opinion and perception of the homegrown school feeding program. Specifically, focusing on the challenges and perceived benefits of a homegrown school feeding program in Addis Ababa.

If you agree to participate in this study, I would like to ask you about the programmatic challenges and perceived benefits of a homegrown school feeding program. It will not take more than 40 to 60 minutes to finish the in-depth interview. I believe there are no risks to you from participating in this study. Also, taking part in this research study may not benefit you personally, but it may help us improve future school feeding program interventions while at the same time improving the nutritional, health, and educational outcomes of primary school students. I appreciate your willingness to take part in this research.

To the best of our ability, we will keep your responses private. When we present this study to others or publish its findings, your name and other identifying information will be removed. Any information that may be used to identify you will be kept separate from your responses, and your responses will be held in a safe location for research purposes only. The data will be saved on password-protected computers.

Participation in this study is voluntary. You have the option of refusing to speak with us or asking us to leave, and if you agree to participate, you can end the interview at any point or skip any questions you do not want to answer. There is no correct or incorrect response. Simply put, we would like to know more about your thoughts and experiences. Whether or not you choose to participate will have no bearing on your job at this institution.

If you have any questions, comments, or complaints, you can reach the researchers through the following addresses: Tel. +251911378982, email: yihalemtamiru@gmail.com, or his supervisor, Dr. Samson Gebremedhin, Tel. +25191682281, e-mail: samsongmgs@yahoo.com.

The Institutional Review Board of Addis Ababa University has reviewed this project. For any concerns regarding this study or if you feel that your rights as a participant (or the rights of another participant) have been violated, please contact the IRB secretary, Dr. Aynadis, by phone during normal business hours at +251912062604 or irb.cncs@aau.edu.et.

Have read and understood the above information. I give consent to participate in the current study.

The names of the key informants whose consent is requested are: _______________

Signature: _______________

Signature of the data collector: _______________ Date:______________

Signature of investigator:__________________ Date: ______________
