# Supplementary material for: Perceived benefits and challenges of school feeding program in Addis Ababa, Ethiopia: a qualitative study
Source: J Nutr Sci. 2024 Sep 18;13:e32. doi: 10.1017/jns.2024.42 (PMC11418071; doi:10.1017/jns.2024.42)
Supplement: Tamiru et al. supplementary material 5 — Tamiru et al. supplementary material [file S2048679024000429sup005.docx]

**Informed Consent for Parents/Guardians of Minors**

**Dear respondent,**

I want to thank you for taking the time to meet with me today. My name is ________, and I would like to talk to you about your opinion and view on the homegrown school feeding program. Specifically, the perceived benefits and challenges of a homegrown school feeding program Your child is being asked to be in a research study of the benefits of the school meal for their educational outcomes, such as attendance, concentration, and academic performance.

The outcomes will be applied to enhance the school meal program even more. Your child will be included in the present study if you permit them to participate in it. The public primary schools in Addis Ababa are the site of this investigation. Your child will be chosen as a potential participant because they are enrolled in the school meal program. Before providing permission for your child to participate in the study, please read this consent form carefully and feel free to ask any questions.

The child's privacy will be safeguarded, and the study's records will remain confidential. The researcher(s) will not include any identifying information in any report that they publish. Information from other participants will be merged with your data. We will write about the combined data we have obtained when we write about the research and discuss the findings with other researchers. Your child's identity won't be revealed in these written documents. The study's findings might be published, but your child's identity and contact details won't be disclosed.

Identifiable research records will be stored securely, and only the researcher(s) will have access to the records. All data will be kept in a locked filing cabinet in the researcher’s office or on a password-protected laptop by the investigator(s). Participation in this study is voluntary and requires your informed consent. Your decision whether or not to have your child participate will not affect your current or future relations with the school. If you decide to have your child participate, they are free to skip any question that is asked. They may also withdraw from this study at any time without penalty.

There are no risks, and your child will not receive compensation or benefits from this research. If you have any questions, comments, or complaints, you can reach the researchers through the following addresses: Tel. +251911378982, email: yihalemtamiru@gmail.com, or his supervisor, Dr. Samson Gebremedhin, Tel. +25191682281, e-mail: samsongmgs@yahoo.com.

The Institutional Review Board of Addis Ababa University has reviewed this project. For any concerns regarding this study or if you feel that your rights as a participant (or the rights of another participant) have been violated or have caused you undue distress (physical or emotional distress), please contact the IRB secretary, Dr. Aynadis, by phone during normal business hours at +251912062604 or irb.cncs@aau.edu.et.

I provide permission for my child to willingly take part in this study by signing below. I certify that I have read and comprehended the study's description. Furthermore, I am aware that I am free to remove my child from the study at any moment without incurring any penalties.

Child’s name:

I do give consent for my child to participate in the study outlined above____________

I do not give my consent for my child to participate in the study outlined by________

Name of the parent/guardian’s for which consent is asked:________ Signature:__________

Signature of the data collector: _______________ Date:____________

Signature of investigator:____________________ Date:_____________
