# Supplementary material for: Perceived benefits and challenges of school feeding program in Addis Ababa, Ethiopia: a qualitative study
Source: J Nutr Sci. 2024 Sep 18;13:e32. doi: 10.1017/jns.2024.42 (PMC11418071; doi:10.1017/jns.2024.42)
Supplement: Tamiru et al. supplementary material 6 — Tamiru et al. supplementary material [file S2048679024000429sup006.docx]

**Interview Guide: HGSFPs Beneficiary Adolescent Grade 6 and 7 Students**

Thank you for meeting with me today. My name is _____, and I would like to talk to you about your experiences and opinions regarding the homegrown school feeding program. Specifically, we are interested in understanding the challenges and perceived benefits of the program in Addis Ababa from the perspective of students in grades 6 and 7. The purpose of our conversation is to gather insights that can help improve future interventions.

This interview will take approximately one hour, and I will be recording our discussion to ensure accuracy. While I will be taking some notes, it's important that you speak clearly to ensure that all your comments are captured. Please remember that your responses will be kept confidential. They will only be shared with the research team, and any information included in our report will be anonymized to protect your identity.

Before we begin, please feel free to ask any questions or seek clarification on anything I've explained. Also, it's essential to understand that your participation is voluntary, and you have the right to decline answering any questions or end the interview at any time.

Do you have any questions or need further clarification before we proceed? Are you willing to participate in this interview?

Thank you for your willingness to contribute to our research.

**Interview Guide: Grade 6 and 7 Students**

**Demographic information**

1. School ……………………………
2. Gender ……………………………
3. Grade ……………………………..

**Perceived Benefits of the Homegrown School Feeding Program:**

1. How has your academic performance been since the program started?
2. In what ways do you think the homegrown school feeding program contributes to school attendance?
3. How do you perceive the effect of the homegrown school feeding program on student dropout rates and absenteeism?
4. What benefits do you believe the program has brought to students like yourself?

**Programmatic challenges and recommendations**

1. Can you identify one aspect of the program that you see as negative?
2. What challenges did you face during the implementation of the home grown school feeding program?
3. What measures or improvements would you suggest to enhance the effectiveness of the homegrown school feeding program in schools?
