# Supplementary material for: Perceived benefits and challenges of school feeding program in Addis Ababa, Ethiopia: a qualitative study
Source: J Nutr Sci. 2024 Sep 18;13:e32. doi: 10.1017/jns.2024.42 (PMC11418071; doi:10.1017/jns.2024.42)
Supplement: Tamiru et al. supplementary material 7 — Tamiru et al. supplementary material [file S2048679024000429sup007.docx]

**Interview Guide: Key Informant Interview for Primary School Directors**

Thank you for taking the time to meet with me today. I appreciate your willingness to participate. My name is _____. The purpose of this interview is to gather your insights and experiences as a primary school director regarding the homegrown school feeding program in Addis Ababa. Specifically, we are interested in understanding the challenges and perceived benefits of the program from your perspective. Our aim is to assess the programmatic benefits and challenges of the homegrown school feeding program for primary school students and stakeholders, in order to gather valuable lessons that can inform future improvements. We are particularly interested in your role as a director and the impact of the program on your school.

The interview is expected to take less than an hour, and I will be recording the session to ensure accuracy. While I will be taking notes, please speak clearly as the session will be taped, ensuring that all your comments are captured. Rest assured, all your responses will be treated as confidential. They will only be shared with the research team, and any information included in our report will be carefully anonymized to protect your identity.

Please remember that your participation is voluntary, and you have the right to decline answering any questions or end the interview at any time. If you have any questions or need further clarification, please feel free to ask. With that in mind, are you willing to participate in this interview? Your valuable insights as a primary school director will greatly contribute to our research. Thank you for your cooperation.

**Interview Guide: School Directors and Coordinators**

**Demographic information**

1. School …………………
2. Age ………….
3. Gender …………..
4. Highest qualification?. Certificate ( ), Diploma ( ), Degree ( ),Master’s degree ( )

**Perceived Benefits of the Homegrown School Feeding Program:**

1. How would you describe the attendance of students since the Homegrown School Feeding Program (HGSFP) started? What do you think has contributed to this change?
2. Have you observed any noticeable differences in students after the implementation of the HGSFP? If so, could you please describe those changes?
3. In your opinion, do you believe the school meal program has had any effect on student behavior in the classroom? If yes, how would you describe that effect?
4. How has the HGSFP improved the educational and health outcomes of the students in your school?
5. In what ways has the HGSFP improved the nutritional status of students? Please provide examples or explain.
6. How do you think the HGSFP will affect school attendance, dropout rates, and academic performance of primary school students?
7. In your experience, how has the HGSFP affected the dropout rates and class repetition of students in your school?
8. To what extent has the home grown school feeding program reduced the financial burden on students' families? Could you please explain?
9. What other changes have you observed in students after the implementation of the HGSFP?

**Programmatic Challenges and Recommendations:**

1. Have you encountered any barriers or challenges in implementing the home grown school feeding program? If yes, could you please describe them?
2. How did you overcome the barriers or challenges faced during the implementation of the home grown school feeding program?
3. What administrative challenges have you and your personnel faced in implementing the homegrown feeding program?
4. In your opinion, what could be possible solutions to tackle the challenges you have identified?
5. Can you outline any measures that are currently in place or that you would suggest to improve the homegrown school feeding program in your school?
