# Supplementary material for: Perceived benefits and challenges of school feeding program in Addis Ababa, Ethiopia: a qualitative study
Source: J Nutr Sci. 2024 Sep 18;13:e32. doi: 10.1017/jns.2024.42 (PMC11418071; doi:10.1017/jns.2024.42)
Supplement: Tamiru et al. supplementary material 8 — Tamiru et al. supplementary material [file S2048679024000429sup008.docx]

**Interview Guide: Key Informant Interview for Stakeholders (Ministry of Education, Sub-City Experts, School Feeding Agency)**

Thank you for taking the time to meet with me today. I appreciate your willingness to participate. My name is ____. The purpose of this interview is to gather your valuable insights and experiences regarding the homegrown school feeding program in Addis Ababa. Specifically, we are interested in understanding the challenges and perceived benefits of the program from your perspective as experts from the Ministry of Education, Sub-City experts, and the Addis Ababa City Administration experts from the School Feeding Agency.

Our aim is to assess the programmatic benefits and challenges of the homegrown school feeding program for different stakeholders, in order to capture important lessons that can inform future program improvements. The interview is expected to take less than an hour, and I will be recording the session to ensure accuracy. While I will be taking notes, please speak clearly as the session will be taped, ensuring that all your comments are captured.

Rest assured, all your responses will be treated as confidential. They will only be shared with the research team, and any information included in our report will be carefully anonymized to protect your identity. You are under no obligation to discuss anything you are uncomfortable with, and you have the freedom to end the interview at any time. If you have any questions or need further clarification, please feel free to ask. I will now proceed with the interview questions. Are you willing to participate in this interview? Your expertise and insights will greatly contribute to our research. Thank you for your cooperation.

**Interview Guide: Stakeholders (Ministry of Education, Sub-City Experts, School Feeding Agency)**

1. Organization ……………
2. Age ………….
3. Gender …………..
4. Highest qualification?. Certificate ( ), Diploma ( ), Degree ( ),Master’s degree ( ), Above( )

**Perceived benefits**

1. How do you anticipate the homegrown school feeding program will impact the lives of students, their families, and the community?
2. In your opinion, will the school lunch program have any effects on student behavior in the classroom? If yes, please explain.
3. How do you expect providing school meals to contribute to the improvement of educational and health outcomes for students? Will they improve, remain the same, or decline?
4. Do you believe the program is currently achieving its intended goals? If yes, please provide reasons for your answer.

**Challenges of the home grown school feeding program**

1. What challenges does your organization or school face in relation to the homegrown school feeding program?
2. In your opinion, what are the potential challenges that schools may encounter in implementing the homegrown school feeding program?
3. Are there any specific areas of the program that you believe should be improved or changed? If so, how would you suggest making those improvements?
4. What actions do you think should be taken to enhance the Homegrown School Feeding Programs in Addis Ababa City?
5. Best regards. Thanks for your time
