# Supplementary material for: Perceived benefits and challenges of school feeding program in Addis Ababa, Ethiopia: a qualitative study
Source: J Nutr Sci. 2024 Sep 18;13:e32. doi: 10.1017/jns.2024.42 (PMC11418071; doi:10.1017/jns.2024.42)
Supplement: Tamiru et al. supplementary material 9 — Tamiru et al. supplementary material [file S2048679024000429sup009.docx]

**Annex 1:** Four-dimension criteria strategies adapted from Lincoln and Guba

**Table 1:** The four-dimensions criteria (credibility, dependability, confirmability, and transferability) strategies adapted from Lincoln and Guba^43^,^53^.

| Rigour Criteria | Purpose | Original Strategies | Strategies applied in our study to achieve rigor |
| --- | --- | --- | --- |
| Credibility | To establish confidence that the results (from the perspective of  the participants) are true, credible  and believable | - Interviewing process and techniques | - Interview protocol was tested before application and pilot interviews were conducted |
|  |  | - Establishing investigators’ authority | - We ensured the investigators had the required knowledge and research skills to perform their roles |
|  |  | - Collection of referential adequacy materials | - We asked interviewers to send all the field notes to the principal investigator for analysis and storage. |
|  |  | - Peer debriefing | - We had regular debriefing sessions with multi-disciplinary co-authors |
| Dependability | To ensure the findings of this  qualitative inquiry is repeatable  if the inquiry occurred within the same cohort of participants,  coders, and context. | - Rich description of the study methods | - We prepared detailed drafts of the study protocol throughout the study. |
|  |  | - Establishing an audit trail | - We developed a detailed track record of the data collection process. Keeping records of the raw data, field notes, transcripts |
|  |  | - Stepwise replication of the data | - We measured the coding accuracy and inter-coders’ reliability of the research team. |
|  |  |  | - ensure the research process is logical, traceable, and documented |
| Confirmability | To extend the confidence that  the results would be confirmed  or corroborated by other researchers | - Reflexivity | - Periodic investigators and coauthors meetings. |
|  |  | - Triangulation | - We applied several triangulation techniques (methodological, data source, investigators, and theoretical). |
|  |  | - Establishing that the researcher’s interpretations and findings | - Reasons for theoretical, methodological, and analytical choices throughout the entire study, so that others can understand how and why decisions were made. |
| Transferability | To extend the degree to which  the results can be generalized or  transferred to other contexts  or settings | - Purposeful sampling to form a nominated sample | - We used a combination of three purposive sampling techniques. |
|  |  | - Data saturation | - We quantified operational and theoretical data saturation. |

**Annex 2:** The sociodemographic characteristics of the study participants are presented in the following Table.

Table 2: Socio-demographic characteristics of study participants

| Participants | Gender | Age range | Number of study participants |
| --- | --- | --- | --- |
| Mother | Female | 25 to 45 | 48 |
| School director | Mixed (F/M) | 25 to 52 | 20 |
| Students | Mixed (F/M) | 12 to 19 | 20 |
| Ministry of education expert | Mixed (F/M) | 35 to 55 | 3 |
| Sub-cities experts | Mixed (F/M) | 30 to 42 | 4 |
| School feeding agency experts | Mixed (F/M) | 32 to 46 | 3 |
| Total study participants | | | 98 |

**Annex 3: Summary of themes and their respective sub-themes**

**Table 3:** Summary of themes and their respective sub-themes

|  | **Themes** |  | **Sub-themes** |
| --- | --- | --- | --- |
| 1 | Perceived benefits of school feeding program | 1 | Improved academic performance, class attendance, attention and, and reduced dropout rates and class repetition |
|  |  | 2 | Reduces the socioeconomic burden of the family |
|  |  | 3 | Improved student behavior and reduced disruptive behavior |
|  |  | 4 | Reducing psychosocial stress and increasing social integrity |
| 2 | Perceived barriers and challenges to homegrown school feeding program | 5 | Underpayment of workers |
|  |  | 6 | The poor market linkage between fostering mothers and consumer cooperatives |
|  |  | 7 | Poor infrastructure |
|  |  | 8 | Increased sense of dependency |
|  |  | 9 | Increase workload for school staff |
|  |  | 10 | Provision of poor-quality food |
|  |  | 11 | Lack of adequate collaboration between the government and stakeholders |
|  |  | 12 | Lack of linkage between SFP and school gardening |
